# Prey capture analyses in the carnivorous aquatic waterwheel plant (*Aldrovanda vesiculosa* L., Droseraceae)

**DOI:** 10.1038/s41598-019-54857-w

**Published:** 2019-12-09

**Authors:** Simon Poppinga, Jassir Smaij, Anna Sofia Westermeier, Martin Horstmann, Sebastian Kruppert, Ralph Tollrian, Thomas Speck

**Affiliations:** 1 Plant Biomechanics Group, Botanic Garden, University of Freiburg, Freiburg im Breisgau, Germany; 2 Freiburg Materials Research Center (FMF), University of Freiburg, Freiburg im Breisgau, Germany; 3 Freiburg Center for Interactive Materials and Bioinspired Technologies (FIT), University of Freiburg, Freiburg im Breisgau, Germany; 4 Department of Animal Ecology, Evolution and Biodiversity, Ruhr-University Bochum, Bochum, Germany; 5 Friday Harbor Laboratories, University of Washington, Seattle, US; 6 Cluster of Excellence livMatS @ FIT Freiburg Center for Interactive Materials and Bioinspired Technologies, University of Freiburg, Freiburg im Breisgau, Germany

**Keywords:** Plant ecology, Plant evolution

## Abstract

We investigated the predator-prey interactions between an Australian ecotype of the carnivorous waterwheel plant (*Aldrovanda vesiculosa*, Droseraceae) and its potential natural prey, the water flea *Daphnia longicephala* (Daphniidae), which also occurs in Australia. *A. vesiculosa* develops snap-traps, which close within ~10–100 ms after mechanical triggering by zooplankton prey. Prey capture attempts (PCAs) were recorded via high-speed cinematography in the laboratory. From 14 recorded PCAs, nine were successful for the plant (the prey was caught), and five were unsuccessful (prey could escape), resulting in a capture rate of ~64%. The prey animals’ locomotion behaviour (antenna beat frequency and movement type) in trap vicinity or inside the open traps is very variable. Traps were mainly triggered with the second antennae. During trap closure, the animals moved only very little actively. A flight response in reaction to an initiated trap closure was not observed. However, several animals could escape, either by having a “lucky” starting position already outside the triggered trap, by freeing themselves after trap closure, or by being pressed out by the closing trap lobes. According to our observations in the successful PCAs, we hypothesize that the convex curvature of the two trap lobes (as seen from the outside) and the infolded trap rims are structural means supporting the capture and retention of prey. Our results are discussed in a broader biological context and promising aspects for future studies are proposed.

## Introduction

The monotypic carnivorous aquatic waterwheel plant (*Aldrovanda vesiculosa* L., Droseraceae) develops snap-traps, a trap type which is otherwise only known from the closely related terrestrial Venus flytrap (*Dionaea muscipula*)^1,2^. However, those of *A. vesiculosa* are markedly smaller in comparison (ca. 2.5–6 mm in length compared to ~2 cm in *D. muscipula*) and capture zooplankton prey in the aquatic medium^3^. *A. vesiculosa* is widely distributed (but nonetheless rare) and naturally occupies habitats in Europe, Asia, Africa, and Australia^4^. Several ecotypes are known, which can be differentiated e.g. by their color^5^. However, prey spectra have only been analysed in plants from Middle-European natural^6^ and naturalized^7^ sites. The results suggest that the waterwheel plant non-selectively captures a wide range of different taxonomic animal groups (including different water flea taxa) of different sizes and with different movement behaviour (substrate-bound, grazer, slow and fast swimmers).

**Figure 1:**
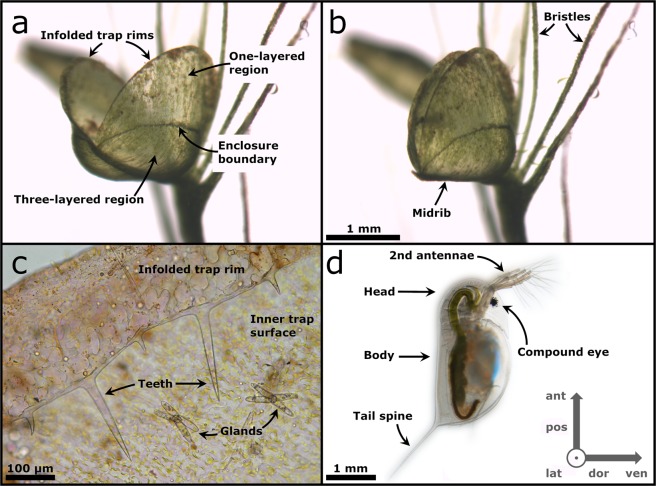
The snap-trap of *Aldrovanda vesiculosa* and the prey daphniid *Daphnia longicephala*. (**a**) A trap in the open condition, prior to triggering. Each lobe consists of a one-layered region, a three-layered region, and an enclosure boundary in between. The infolded trap rim is visible (see also (c)). The trigger hairs are situated inside the trap close to the midrib and are not visible in this image. The scale bar in (b) also applies for (a). (**b**) The trap in the closed condition, after triggering manually with a fine nylon thread. The two trap lobes are connected by a midrib. Foliar bristles are indicated. (**c**) Light microscopy image of the infolded trap rim. The trap-inward orientated teeth are visible. The inner trap surface possesses a multitude of glands. (**d**) General morphology of *Daphnia longicephala*. The head with second antennae and the compound eye as well as the tail spine are indicated. Body directions are given in the coordinate system: ant = anterior, dor = dorsal, lat = lateral, pos = posterior, ven = ventral.

The *A. vesiculosa* trap consists of two convex lobes (as seen from the outside), which are connected by a midrib (Fig. 1a,b). The central trap lobe part forms the so-called three-layered region (consisting of two epidermal cell layers on either side of a parenchymatous layer), and the upper part the single-layered region (consisting of two epidermal layers). Both regions are connected by an enclosure boundary^8^. The trap lobes have infolded rims with teeth-like protrusions (Fig. 1a,c). Mechanical stimulation by prey on trigger hairs, which are primarily located in great number in the central region inside the trap below the enclosure boundary, entails the trapping movement of *A. vesiculosa*. The two trap lobes rapidly move toward each other by a combination of hydraulic actuation^8,9^, kinematic amplification by curved folds^10^, and the release of pre-stress in the midrib^11^ until the trap is shut. The trap closure duration can be as short as 10 ms^11^. Therefore, the *A. vesiculosa* trap ranks among the fastest motile traps in carnivorous plants. Even faster are aquatic bladderworts (*Utricularia* spp., Lentibulariaceae) with suction traps, which are comparable in size and prey spectra to *A. vesiculosa* snap-traps. The Southern bladderwort (*U. australis*), which occasionally co-occurs with *A. vesiculosa*^6,7^, can engulf its prey within 5.2 ms at fastest (9 ms on average)^12^.

The exact procedure of prey capture of *A. vesiculosa* has not yet been investigated. Hence, knowledge is very scarce or absent regarding the motion sequences of the trap during capture, the prey behaviour while approaching, entering and triggering the trap, as well as conceivable escape attempts by prey. Detailed analyses of the predator-prey interaction would allow for a better general understanding of trophic interactions and possible aspects of predator-prey coevolution. Therefore, we investigated the interactions between an Australian ecotype of the carnivorous waterwheel plant and its potential natural prey, the water flea *Daphnia longicephala* (Daphniidae) (Fig. 1d).

## Results

### General results

We recorded 14 high-speed movies of prey capture attempts (PCAs 1–14, Movie 1–14), i.e. prey-triggered trap closures. Details of the general results are presented in Table 1. As also noted in the Materials and Methods section, PCAs were grouped according to their successful or unsuccessful outcome, so that the sequence presented (PCAs 1–14) is not in the same chronological order as in the actual experiments. In PCAs 1–9, the respective traps successfully captured the prey animals. In the other five PCAs 10–14, the prey animals triggered the traps but were not caught. This corresponds to a capture rate of ~64%. No correlation between prey density in the cuvettes (values in Table 1, see also Materials and Methods for details) and prey strike outcome (successful or unsuccessful) could be detected (biserial test: 0.2793872; spearman rho = 0.214004; p = 0.4626). Prey swam into the respective traps without performing any conspicuous activities. Any contact between trap and animal appeared to happen coincidentally. Prey triggered the trap always by touching the trigger hairs situated on the inner trap surface beneath the enclosure boundary (i.e. in the central region).

**Table 1:** Detailed results of the recorded PCAs.

PCA	Successful prey capture	t_trigger_	t_DZ_ [ms]	t_TI_ [ms]	t_closure_ [ms]	Prey density [n/ml]
1	✓	10 min	321	179	17	~3
2	✓	2 min	56	208	14	~5
3	✓	15 min	399	659	16	~3
4	✓	4 min	176	1,411	21	~8
5	✓	10 min	186	434	45	~8
6	✓	15 min	1,083	415	26	~8
7	✓	10 min	73	2,362	77	~8
8	✓	<10 s	—	—	17	~7
9	✓	1 min	293	199	—	~5
10	×	30 min	459	382	16	~5
11	×	15 min	276	291	18	~5
12	×	1 min	171	846	16	~8
13	×	<10 s	55	758	30	~3
14	×	6 min	382	419	104	~5

PCAs were grouped according to their successful or unsuccessful outcome, so that the sequence presented is not in the same chronological order as in the actual experiments. Abbreviations: t_trigger_ = time span from the introduction of the prey until the triggering of the trap; t_DZ_ = staying duration of prey inside the danger zone; t_TI_ = staying duration of prey in the trap interior; t_closure_ = duration of trap closure ( = trap snapping duration).

The shortest time span from the introduction of the prey into the cuvettes until the prey triggered the respective trap (t_trigger_) was less than ten seconds (PCAs 8 & 13). Due to the experimental handling procedures, a more precise indication is not possible. The longest t_trigger_ is approximately 30 minutes (PCA 10). Between these minimum and maximum values, t_trigger_ varies greatly. The result of the Shapiro-Wilk test with p = 0.03 (for n = 14) shows that the data are not normally distributed. The median is 480 s. We could not detect a correlation between prey density and t_trigger_ (spearman rho = 0.02805953; p = 0.9241).

**Figure 2:**
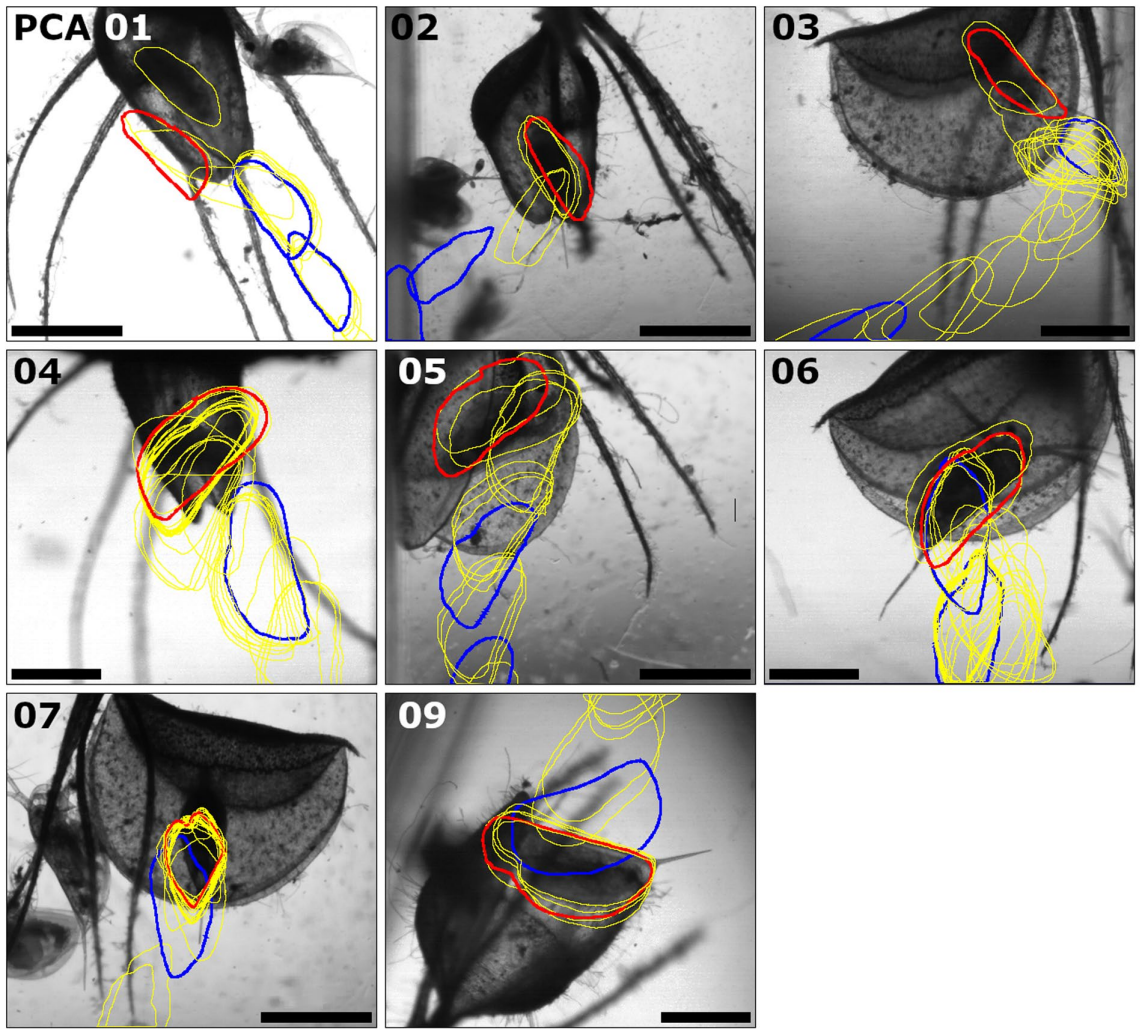
Movement profiles of *Daphnia longicephala* during the successful PCAs 1–7 & 9. The images show the redrawn contours (in yellow) of the respective prey animals, with the positions marked in blue when entering the danger zone and the trap. The contour of the position before the prey triggers the trap is marked in red. The images shown are the last images of the respective PCA high-speed recordings (Movies S1–7 & 9), depicting the final recorded position of the prey inside the closed trap. For PCA 8, no movement profile could be created (cf. the general results section). The orientation of the prey towards the midrib at the time when the respective trap was triggered is indicated in Table 2. The scale bars are 1 mm. The respective high-speed movies have been acquired by the IDT Motion Studio software (v.2.10.05) and were further processed with the Fiji/ImageJ software, see Materials and Methods for details.

The results for the time spent by prey in the danger zone (t_DZ_, see Materials and Methods for definition) also scatter over a wide range. The shortest value measured for t_DZ_ is 55 ms (PCA 13), whereas the longest is 1,083 ms (PCA 6). The Shapiro-Wilk test showed that the t_DZ_ results are not normally distributed (p = 0.003, n = 13). The median is 276 ms. In PCA 8, the prey was already situated inside the trap at the beginning of the recording, without making large movements, so that no t_DZ_ could be detected in this case (cf. Figure 2 where PCA 8 does not occur).

For the reasons mentioned above, also for the time spent by prey inside the trap before triggering (t_TI_) no value could be determined for PCA 8. For the remaining PCAs, the results for t_TI_ vary greatly. The lowest value is 179 ms (PCA 1), the highest is 2,362 ms (PCA 7). The data is not normally distributed (p = 0.002, n = 13). The median for t_TI_ is 419 ms.

In PCA 9, the prey is clamped between the trap lobes and the trap closure duration ( = trap snapping duration, t_closure_) could therefore not be measured. Trap closure durations are comparably high in PCA 5 (t_closure_ = 45 ms), PCA 7 (t_closure_ = 77 ms), and PCA 14 (t_closure_ = 104 ms), where the respective traps had comparatively many filamentous algae attached to their surfaces. Except for these outliers, all other measured values for t_closure_ are between 16 ms and 30 ms and are not normally distributed (p = 0.019, n = 10). The median for t_closure_ (including the above-mentioned outliers) is 18 ms.

### Prey animal movements and trap triggering events during PCAs

Detailed results on the prey animal movements are presented in Table 2. The total numbers of prey movements during the respective PCAs (n_prey_movement_) vary between two (PCA 8) and 20 (PCA 3), with a median of 8 and a mean of 9 ± 4. No correlation between n_prey_movement_ and capture success could be determined (biserial test: 0.09183631; spearman rho = -0.01897583; p = 0.9487). The age of the prey does not correlate with the number of prey movements (spearman rho = 0.07811666; p = 0.7998), whereby an age of 5.5 days was defined for PCA 2. Here, due to an accidental mixing of prey animals of 5 and 6 days of age, the exact age could not be defined. PCA 13 was excluded from the calculation of the correlation coefficient due to unknown age of the prey. Also, no correlation between the age of the prey and the outcome of the prey strike (successful or unsuccessful) could be determined (biserial test: −0.08887876; spearman rho = −0.1278854; p = 0.6772).

**Table 2:** Detailed results of prey movements during PCAs.

PCA	n_prey_movement_	n_antenna_return_stroke_	n_antenna_downstroke_	n_antenna_asynchronous_	Prey position and orientation during trap triggering: 1) Position inside the trap 2) Body orientation relative to midrib 3) Orientation of head	Trap triggering by:	Prey age
**1**	8	4	3	1	1) One-layered region 2) Inclined, more or less orthogonal 3) Trap-inwardly	Second antennae return stroke	3 days
**2**	5	2	1	2	1) One-layered region 2) Orthogonal 3) Trap-inwardly	Asynchronous second antennae movement	5-6 days
**3**	20	6	6	8	1) Between one- and three-layered region 2) Orthogonal 3) Trap-inwardly	Second antennae downstroke	3 days
**4**	11	2	3	6	1) Trap margin 2) Transverse; ventral side is oriented trap-inwardly	Asynchronous second antennae movement	6 days
**5**	8	3	4	1	1) Between one- and three-layered region 2) Oriented obliquely along midrib 3) Trap-outwardly	Second antennae downstroke	6 days
**6**	10	3	3	4	1) One-layered region 2) Oriented obliquely along midrib 3) Trap-inwardly	Asynchronous second antennae movement	6 days
**7**	8	2	1	5	1) Trap margin 2) Transverse; ventral side is oriented trap-inwardly	Asynchronous second antennae movement	6 days
**8**	2	1	1	–	1) Between one- and three-layered region 2) Inclined, more or less orthogonal 3) Trap-inwardly	Second antennae return stroke	4 days
**9**	11	3	4	4	1) Trap margin 2) Transverse; ventral side is oriented trap-inwardly	Second antennae downstroke	6 days
**10**	8	4	3	1	1) Trap margin, partly outside of trap 2) Orthogonal 3) Trap-inwardly	Asynchronous second antennae movement	6 days
**11**	12	5	4	3	1) Trap margin; almost completely outside of trap 2) Orthogonal 3) Trap-outwardly	Tail spine wobbling	3 days
**12**	8	1	1	6	1) Trap margin 2) Transverse; lateral body sides are oriented trap-in- and outwardly	Asynchronous second antennae movement	6 days
**13**	5	2	1	2	1) Trap margin 2) Parallel; ventral side is oriented trap-outwardly	Second antennae return stroke	unknown F1
**14**	10	3	3	4	1) Trap margin; during the onset of trap closure, the prey is completely outside of the trap 2) Parallel; ventral side is oriented trap-inwardly	Water movement caused by second antennae downstroke	6 days

PCAs were grouped according to their successful or unsuccessful outcome, so that the sequence presented is not in the same chronological order as in the actual experiments. Abbreviations: n_prey_movement_ = total number of prey movements during the respective PCA; n_antenna_return_stroke_ = number of second antennae return strokes during the respective PCA; n_antenna_downstroke_ = number of second antennae downstrokes during the respective PCA; n_antenna_asynchronous_ = number of asynchronous second antennae movements during the respective PCA.

**Figure 3:**
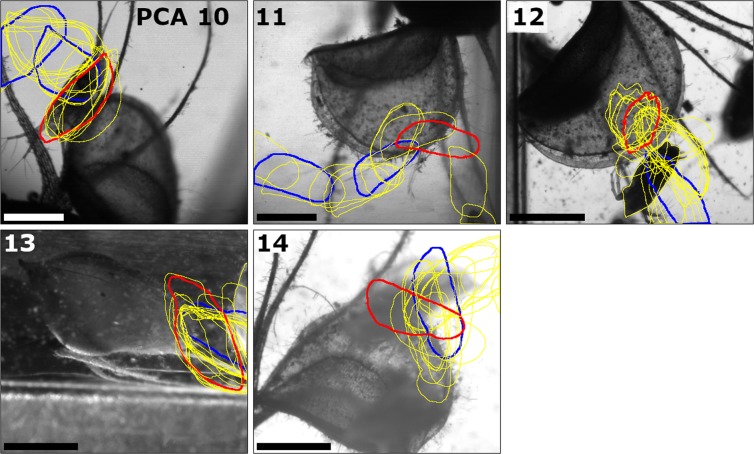
Movement profiles of *Daphnia longicephala* during the unsuccessful PCAs 10–14. The images show the redrawn contours (in yellow) of the respective prey animals, with the positions marked in blue when entering the danger zone and the trap. The contour of the last position before the prey triggers the trap is marked in red. The images shown are the last images of the respective PCA high-speed recordings (Movies S10–14), showing the final recorded position of the prey inside the closed trap. The orientation of the prey towards the midrib at the time when the respective trap was triggered is indicated in Table 2. The scale bars are 1 mm. The respective high-speed movies have been acquired by the IDT Motion Studio software (v.2.10.05) and were further processed with the Fiji/ImageJ software, see Materials and Methods for details.

The movement profiles of *D. longicephala* during PCAs 1–7 & 9–14 are depicted in Figs. 2 and 3. For the same reasons mentioned in the general results section, it is not possible to depict PCA 8 in this manner. Detailed results for prey positions and orientations are shown in Table 2. Eight animals (~57%) were situated at the trap margins during the respective trap triggering events, three (~21%) within the one-layered regions, and three (~21%) between the one- and the three-layered regions. Five animals (~36%) were oriented orthogonally in relation to the respective midrib with trap-inward oriented heads. In one orthogonally oriented animal (~7%), the head was facing trap-outwards. Three animals (~21%) were oriented transversely in relation to the midribs, with their ventral sides facing trap-inwards. In one transversely oriented animal (~7%), the lateral body sides were oriented either trap-in- or outwardly. Two animals were oriented obliquely along the midrib, one with its head facing trap-inwards, one with its head facing trap-outwards (~7% each). Two animals were oriented parallel to the midribs, one with its ventral side facing trap-inwards, one with its ventral side facing trap-outwards (~7% each).

In 12 out of the 14 PCAs (~86%), the prey animals triggered the traps directly with one of their second antennae. In one case (~7%), the prey triggered the trap with its tail spine (PCA 11) and in one case (~7%) indirectly by the water movement caused by a downstroke of the second antennae (PCA 14). From the 12 triggering events directly caused by movements of the second antennae, in three cases (25%) the respective movements were return strokes, in another three cases (25%) downstrokes, and in six cases (50%) asynchronous movements.

**Figure 4:**
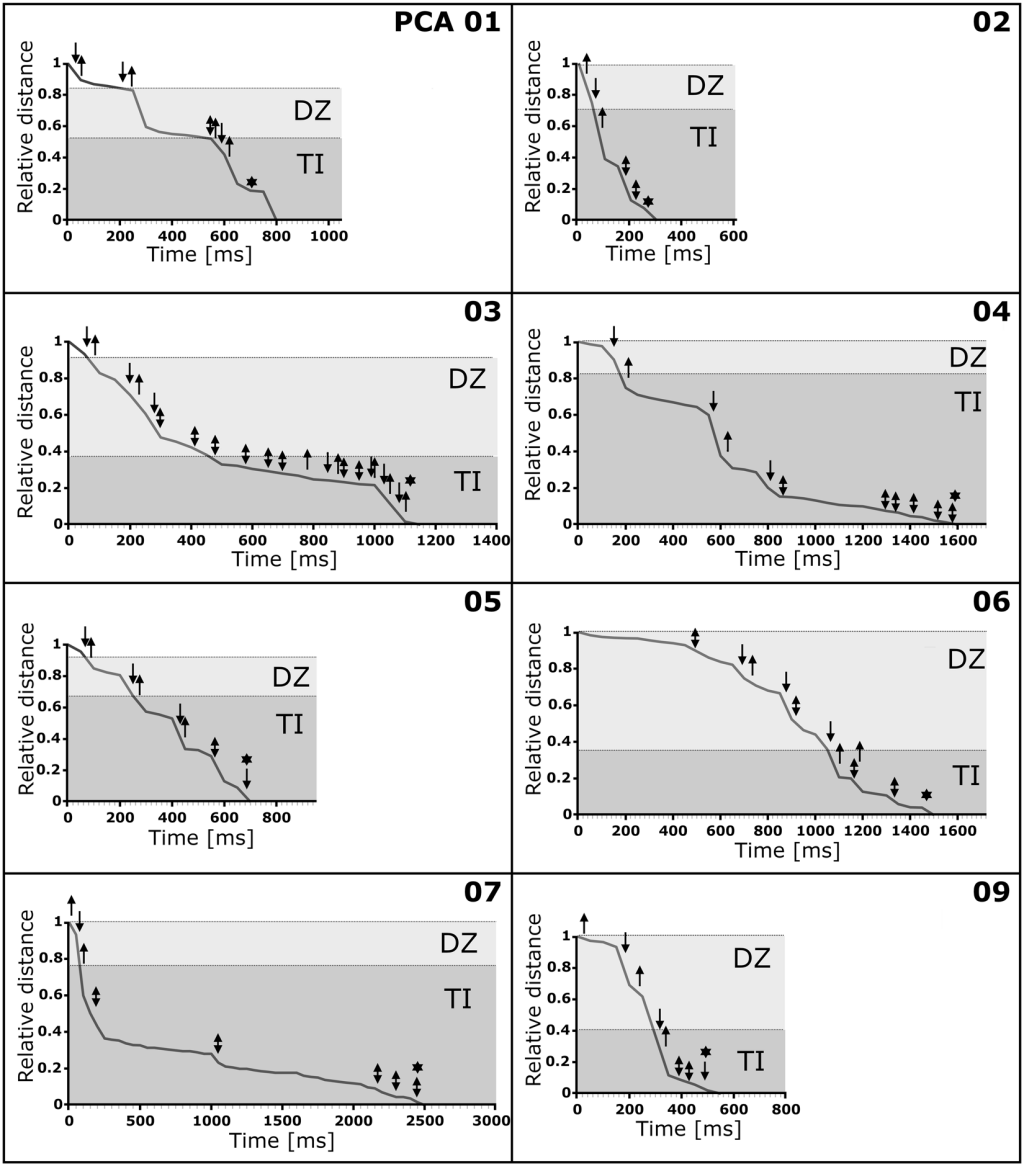
Graphical representation of *Daphnia longicephala* movements and behaviour during the successful PCAs 1–7 & 9. The relative distances of the prey animals to their respective final positions (see Materials and Methods for details) are indicated. The danger zones (DZ) are depicted in light grey, the trap interior zones (TI) in dark grey. The following events are indicated by symbols: an upwards pointing arrow corresponds to a second antennae return stroke, an arrow pointing downwards corresponds to a second antenna downstroke, a double arrow corresponds to an asynchronous second antennae movement, and an asterisk corresponds to trap triggering.

Graphical representations of prey movements and behaviour during the successful PCAs 1–7 & 9 are shown in Fig. 4 (detailed results are presented in Tables 1 and 2). The values for t_DZ_ and/or t_TI_ as well as the types and amount of movements vary greatly between the different successful PCAs. For example, the prey animal in PCA 2 briefly (t_DZ_ + t_TI_ = 264 ms) swam through the danger zone (DZ) into the trap with executing only few movements (n_prey_movement_ = 5) until it was finally caught. In contrast, the prey animal in PCA 3 approached the trap and swam into it much slower in direct comparison (t_DZ_ + t_TI_ = 1,058 ms) and with executing many more movements (n_prey_movement_ = 20). Especially in the trap interior (TI) the prey executed multiple movements (14) without triggering the trap. For PCA 7 it can be seen that the prey passed the DZ in short time (n_DZ_ = 73 ms) with only three second antennae strokes. In the FI the prey animal executed five movements (asynchronous movements of the second antennae) and it took a comparably long time until the trap was triggered (t_TI_ = 2,362 ms). Both PCAs 4 & 6 showed approximately the same duration (PCA 4: t_DZ_ + t_TI_ = 1,587 ms; PCA 6: t_DZ_ + t_TI_ = 1,498 ms). However, the prey in PCA 4 spent most of its time in the TI and executed most of its movements there, whereas the prey in PCA 6 spent only a short time in the TI and executed most of its movements in the DZ.

In three out of the nine successful PCAs (5, 7 & 9), the prey was clamped between the trap lobes in such a way that the otherwise fast snapping motion was slowed down. In PCA 5, the infolded spiny trap rim (cf. Fig. 1a,c) acts as a barrier, potentially facilitating prey clamping and preventing its slippage out of the trap. Due to the pressure exerted by the moving lobes in PCA 7, the clamped prey turned around and then was forced deeper into the trap. In the three other successful PCAs (3, 4 & 6), a body part of each trapped prey animal was stuck between the lobe margins and protruded from the trap. In PCA 3 it was the tail spine, in PCA 4 one of the second antennae, and in PCA 6 the rear end of the body including the tail spine. Within a few minutes after recording, all prey animals were completely inside the closed traps.

**Figure 5:**
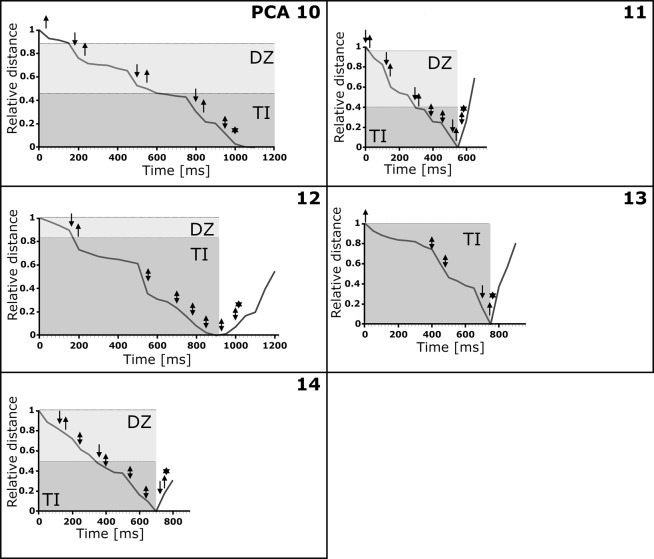
Graphical representation of *Daphnia longicephala* movements and behaviour during the unsuccessful PCAs 10–14. The relative distances of the prey animals to their respective final positions (see Materials and Methods for details) are indicated. The danger zones (DZ) are depicted in light grey, the trap interior zones (TI) in dark grey. The following events are indicated by symbols: an upwards pointing arrow corresponds to a second antennae return stroke, an arrow pointing downwards corresponds to a second antenna downstroke, a double arrow corresponds to an asynchronous second antennae movement, and an asterisk corresponds to trap triggering. Due to the altered geometry of the closed trap after triggering, a discrimination between DZ and TI was not possible during the escape of the prey animals. In PCA 10, the prey could free itself only after the end of the recording.

Graphical representations of prey movement and behaviour during the unsuccessful PCAs 10–14 are shown in Fig. 5 (detailed results are presented in Tables 1 and 2). In the cases of PCAs 11–14, the increases of the relative distances in the graphs towards the ends of the recordings depict the prey animals’ flight out of the respective traps. This is not the case in PCA 10, where the prey was partially stuck in the trap, but was able to free itself later after the recording. In PCAs 10, 13 & 14 the relative distances rise steeply towards the end, as depicted in the graphs, which corresponds to fast swimming responses of the animals. The respective traps started to close after the beginning of these swimming motions. In PCA 12, the relative distance first increases comparatively slowly, when the prey moved away from the midrib. When the trap was triggered, the prey was clamped for a short period, which corresponds to the flattening of the graph in the graphical representation. After the prey freed itself, it fled with fast motions, leading to a steep rise of the relative distance in the respective graph.

In the five unsuccessful PCAs 10–14, an active escape of the respective daphniid could only be observed in two cases. During the recording of PCA 10, the second antennae of the animal were clamped between the infolded trap rims. However, after the end of the recording the prey could free itself. In PCA 12, the prey was clamped transversely (in relation to the midrib) within the one-layered region, but managed to free itself by an asynchronous second antenna strike. In the remaining three unsuccessful PCAs, the prey was already swimming out of the trap, thereby performing motions which triggered the respective trap closure events. In PCA 13, the prey was initially (slightly) orientated with its head towards the inside of the trap. However, during the return stroke of the second antennae, which triggered the trap, it had already turned and was oriented with its dorsal side towards the trap. The moving trap lobes, which touched the animal, glided off from the slight wedge-like shape of the carapace and pushed the prey further out.

## Discussion

### Trapping efficiency

In most studies on the functional principles of carnivorous plant traps, the respective experiments and kinematical analyses were performed with artificially triggered traps^10,11,13,14^. There are only few reports available, which (in some cases only superficially) deal with the interrelation of prey and carnivorous plant trap movements^15–18^. A detailed report on the ultra-fast underwater suction traps of the aquatic carnivorous plant *Utricularia australis* (Lentibulariaceae) and its daphniid prey *Ceriodaphnia dubia* was published recently^12^. In this study, which is methodically similar to the present one, the processes of mechanical trap triggering by the prey, trapdoor opening, suction of water and prey, and trapdoor closure have been recorded with high temporal resolution. The sequence of rapid motions (partly in the sub-millisecond regime) performed by the *Utricularia* suction trap and the resulting high acceleration of the sucked prey, its sudden and abrupt deceleration inside the trap, as well as the fine-tuned trapdoor closure for prey retention, strongly suggest that *C. dubia* has no chance to escape the trap once it is triggered. At least, all 14 prey capture attempts recorded in this *Utricularia* study were successful.

In the present study on *A. vesiculosa* and *D. longicephala*, also 14 prey capture attempts were analysed, with a lower resulting capture rate (~64%). The measured *A. vesiculosa* trap closure durations typically ranged between 16–30 ms (longer durations also occurred in traps with many attached filamentous algae), in general corroborating earlier snapping duration measurements^8,11^ and exceeding the duration of suction in *Utricularia* (9 ms on average, min: 5.2 ms; max: 14.9 ms, n = 14)^12^. Together with the escape of several animals, as observed in this study, one may suggest that prey is more likely to escape the *A. vesiculosa* snap-trap than the *U. australis* suction trap. However, this assumption has to be taken with caution, as two different prey animal species were tested under similar, but not identical experimental laboratory conditions. Furthermore, the sample sizes are small (n = 14 each). However, a low capture efficiency has also been attributed to *A. vesiculosa* by other authors, based on the fact that during field studies only 5–11.5% of the investigated traps contained prey^6,19^. To what extent these field observations indeed reflect the efficiency of the snap-trapping mechanism and/or the efficiency of prey attraction is not yet clear and remains a promising subject for future studies. Interestingly, a low capture efficiency has also been reported for Venus flytraps (*D. muscipula*), the terrestrial sister of *A. vesiculosa*, in several field- and lab-based studies^20–22^.

### Trap irritability

The trigger hairs are situated in the central trap lobe parts beneath the enclosure boundary. The observation in PCA 14 that prey movement-induced water displacement triggers the trap hints towards a high sensitivity of the *A. vesiculosa* sensory system. A very sensitive triggering mechanism on the one hand presumably enhances the efficiency of the trap by enabling it to detect also slight mechanical perturbations. But on the other hand, it also requires “safety precautions” for avoiding energy demanding and unnecessary trap closures e.g. by water streams occurring in the habitat. In this context, the trigger hair occurrence at the trap base might be a structural measure of precaution for avoiding unnecessary trap closures, as the hairs are protected from water streams and detritus. It is also conceivable that it increases the capture efficiency because potential (small) prey will be inside the trap when the trigger hairs become stimulated. However, in this study with *D. longicephala*, which possesses relatively long appendages like the second antennae and the tail spine, we observed that these structures were often responsible for trap triggering, without the prey animal’s body being necessarily fully inside the trap. Future studies could analyse the “suitability” of prey and the trap irritability^23,24^ in greater detail. A highly sensitive sensory system was recently described for the snap-trap of the waterwheel’s sister, the Venus flytrap (*D. muscipula*)^25,26^. Whereas in *D. muscipula* there are always at least two consecutive stimuli on one or on different trigger hairs necessary within a certain period to entail trap closure, the traps of *A. vesiculosa* are more variable in this respect. Here, some traps close after reception of one stimulus, some close after two stimuli, some require even more mechanical perturbations, and some do not respond at all^1,2,8^. The reasons for this variability in trap irritability are still unknown.

### Possible mechanisms of prey attraction

It has been hypothesized that the foliar bristles situated next to the trap of *A. vesiculosa* (Fig. 1b) could attract animals by providing them a place to rest and protection^27^. The numerous trigger hairs inside the snap-trap (cf. Fig. 3c in ref. ^11^) were discussed to mimic filamentous algae in order to lure grazing crustaceans into the snap-trap^28^, but clarifying studies are missing so far. Furthermore, since Middle European *A. vesiculosa* shows no prey specificity but catches opportunistically, independent of prey species, size, mobility mode, and speed of movement, it is unlikely that the capture is enhanced by specialized chemical- or mimicry-based attraction mechanisms^7^. From the erratic swimming behaviour by *D. longicephala* we could not discriminate and observe any behaviour indicating attraction towards the trap. Since *D. longicephala* is a good and active swimmer, with an average swimming velocity of 8 mm/s, the probability of an encounter with an *A. vesiculosa* trap is probably high^29,30^. Therefore, it is conceivable that natural PCAs happen rather coincidentally, probably e.g. because the animals approach the trap during the search for food.

Most traps in the recorded PCAs 1–14 were oriented downwards due to methodical/handling reasons (Figs. 1 and 2). However, the trap leaves of *A. vesiculosa* are arranged as whorls (cf. Fig. 1b in ref. ^11^) and, therefore, their natural orientation is variable. We hypothesize that the orientation of the traps does not play a crucial role in the possible attraction and capture of free-swimming prey like *D. longicephala*. In this context, it could be interesting to investigate the effect of trap orientation on substrate-bound or grazing prey.

### General aspects of prey movement and behaviour

Due to their wide scattering, the results obtained for t_DZ_ and t_TI_ do not reveal typical staying times within the different trap zones. In addition, the types and amount of movements vary greatly between the different PCAs. It appears to be purely coincidental how long the prey stays in the danger zone and/or in the trap interior prior to triggering. Also in the Southern bladderwort (*U. australis*) with suction traps, the body orientation and movement behaviour of the prey daphniid *C. dubia* were variable before and during triggering^12^.

In 12 cases in this study (~86%), the animals triggered the *A. vesiculosa* traps directly with movements of their second antennae. These comparably long structures protrude from the animal’s head and are responsible for swimming movements by generating thrust. Regardless of most observed orientations and positions of the prey animals at the events of trap triggering (cf. Table 2), the second antennae came into contact with the trigger hairs inside the traps (PCAs 1–10, 12,13), or generate strong enough water currents for triggering (PCA 14). Similar observations are reported in the already mentioned *Utricularia* prey capture study^12^, where seven (out of 14) *Ceriodaphnia dubia* animals touched the trigger hairs with their second antennae, six with their heads, and one with its carapace. We may speculate that the second antennae are the main structures with which daphniid prey come into contact with the trigger hairs of aquatic carnivorous plants’ traps.

The profiles of *D. longicephala* movements during the PCAs (Figs. 2 and 3) show that in most cases, successful prey capture is accompanied with very little prey movement inside the closing trap. This is in contrast to *U. australis*, where the prey is often passively turned around and may even loop during capture due to the forces of the water influx^12^. In *A. vesiculosa*, the two trap lobes clasp around the prey without further relocating it to a great extent, e.g. by induced water movement. How *A. vesiculosa* is able to capture and retain animals larger than the actual traps, as e.g. chironomid pupae or notonectid nymphs^6,7^, remains to be investigated. Probably, the teeth on the infolded rim (Fig. 1a,c) and/or ongoing slow processes of trap deformation, i.e. stomach formation^8^, help in retaining and enclosing such large objects.

### Escape of prey during unsuccessful PCAs

There are no reports available on prey escape attempts from triggered *Utricularia* traps. Apparently, the *Utricularia* trap entrance, where also the trigger hairs are situated, is a “point of no escape” due to the fast onset of strong suction (at least as long as the prey fits through the entrance and into the trap). On the contrary, in this study with *A. vesiculosa* we observed five unsuccessful PCAs (10–14), indicating that prey animals situated at the region of triggering (the central lower trap part below the enclosure boundary) still have a chance to escape. We observed that prey could escape either due to the forcible pulling of its clamped body (or body parts) out of already closed traps (PCAs 10, 12), or due to the fact that trap lobes with teeth on the infolded rims (Fig. 1a,c) may glide off the respective carapace during trap closure (PCA 13). Prey, which was already swimming out of the trap during triggering, had a favourable “starting position” and could escape as well (PCAs 11, 14). This shows that at least three different trap triggering/movement situations exist where prey can potentially flee. However, fully enclosed prey animals never escaped. A true flight response, i.e. an animal actively swimming out of a closing trap as a response to the triggered motion, was not observed in this study with *D. longicephala*. We consider it indeed as unlikely that daphniids in general are able to respond in the required timescale between trap triggering and closure. This is also based on the fact that part of the authors of this study (SK, MH, RT) could also not observe any flight response of *Daphnia pulex* that were captured by larval phantom midges (Diptera: *Chaoborus*), where the actual strike of the predator lasts ~30 ms and the whole capture process less than 300 ms^31^.

The two escape events of clamped prey (PCAs 10 & 12) show that *D. longicephala* is able to mechanically resist the force of the closing trap lobes of *A. vesiculosa* and to free itself by antenna strokes. Besides the age and condition of the trap as well as the environmental conditions as determining factors, the closing force has been shown to increase with increasing triggering stimulus strength^8^. How clamped prey is further “processed” by the trap may also be influenced by the position of the animal and its physical condition. In PCA 7, for example, the prey was initially clamped diagonally between the lobes (similar to PCA 12). Then the prey turned around, presumably purely passively due to the force of the two trap lobes acting on it. Subsequently, the curved lobes closed further and pushed the prey deeper into the trap until the trap was fully closed. Accordingly, it may be speculated that the doubly curved geometry of the lobes, which does not change during trap closure^10^, helps in “handling” of prey clamped between the one-layered regions of the lobes. Additionally, as observed in PCA 5, the infolded spiny trap rim may help in retaining prey. However, as discussed for PCA 13, it may also happen that the trap rim glides off during the capture motion. In the closely related Venus flytrap (*D. muscipula*), where the teeth are much longer in relation to the overall trap dimension compared to *A. vesiculosa*, it has initially been hypothesized^32^ and later found in a field- and lab-based study^22^ that the teeth form a “prison” that increases prey capture success for moderate-sized prey. However, for larger prey a decreasing benefit was also found, which adds to the complexity of the adaptive landscape of the snap-trap system.

### Critical evaluation of applied methods

Older *A. vesiculosa* traps have different physiological characteristics than younger ones^8^. In order to ensure that all traps used in the experiments are of similar age, only traps of the seventh whorl were chosen. As the Australian red ecotype of *A. vesiculosa* develops 0.26–0.65 trap leaf whorls per day^33^, the ages of the respective traps presumably scatter between 11–27 days accordingly. However, we may speculate that individual differences between the traps, such as their sizes or the amount of attached algae, possibly influence their snapping behaviour (e.g. irritability, kinematics, snapping speed) to a greater extent than the (small) age differences. For example, in our study the highest snapping duration of 104 ms was measured in a trap with many attached algae (PCA 14).

The experimental conditions in the climate-constant room under artificial light self-evidently deviated from the natural habitat conditions of Australian *A. vesiculosa* and *D. longicephala*. For example, the small volumes of the cuvettes restricted prey and water movement. Due to the illumination, the water column in the cuvettes probably warmed up (not measured). The illumination presumably irritated the daphniid prey. Furthermore, we cannot exclude that cutting the traps off the respective plants leads to unwanted (and unnoticed) side-effects, evoked e.g. by stress responses and ultimately leading to an altered trap behaviour and a biased trapping efficiency evaluation. The high-speed analyses including the handling of the delicate and sensitive structures (like the open, ready-to-catch *A. vesiculosa* traps) are challenging and time-consuming tasks and explain the small sample size in this study. However, regardless of these drawbacks inherent to our experimental approaches, our study is the first attempt to shed light on predator-prey interaction between the carnivorous plant *A. vesiculosa* and its prey *D. longicephala*. Such high-speed analyses of fast aquatic organisms and structures would otherwise be very difficult (or even impossible) to perform in the field under natural conditions.

### Conclusion and outlook

Our study is the first to show in detail how the carnivorous waterwheel plant (*A. vesiculosa*) captures its daphniid prey. The movement sequences of its underwater snap-traps as well as the *D. longicephala* behaviour and motions outside and inside the traps are described qualitatively and quantitatively. Additionally, cases of movement interrelations (e.g. prey escapes, handling of clamped prey by the trap) are described and discussed for the first time.

We could not detect correlations (1) between prey density and capture success, (2) between the age of prey and capture success, (3) between n_prey_movement_ and the capture success, (4) between prey density and t_trigger_, and (5) between the age of prey and n_prey_movement_. Our analyses also show that the predator-prey interaction as observed here is by no means uniform. Individual differences in prey behaviour as well as between the individual traps apparently dominate. By assuming even greater kinematic differences between older and younger traps, even more inhomogeneous situations regarding prey capture sequences may emerge. Future experiments could complementarily analyse PCAs with different natural prey animals, e.g. members of Cladocera, Copepoda, Ostracoda, Ephemeroptera, Nematocera, Hydrachnidia, and Pulmonata^7^. Such analyses would be helpful for further evaluating the capture efficiency of the *A. vesiculosa* traps, which feed on a very high diversity of prey animals regarding taxonomy, size, and movement behaviour. Therefore, a broad spectrum of *A. vesiculosa* trap (motion) characteristics could possibly be a selective advantage. A similar non-selective prey capture behaviour has been reported from adult traps of the Venus flytrap (*D. muscipula*)^32,34^. Interestingly, traps of *D. muscipula* seedlings capture different prey, which apparently are too small to trigger the adult traps^35^. Such potential prey specificity at different growth stages has not yet been reported for *A. vesiculosa*. Future kinematic prey capture investigations should also include elaborate 3-dimensional movement analyses^30,36^.

Other possible topics for future studies are the developmental and structural-biomechanical characteristics of possible counter-measures by the prey against capture by *A. vesiculosa*. For example, several daphniids are known to possess predator-specific inducible defences, which may comprise alterations in morphology, behaviour, and life history^37–39^. With these measures, the prey is capable to thwart the predator and to reduce predation pressure. Therefore, it would be interesting to test with typical daphniid prey of *A. vesiculosa* if such inducible defences also exist in the here described plant predator - animal prey relationship. Especially analysing the curvature of the carapace, which we observed to influence how ‘easy’ a half-trapped animal is forced inside or outside the trap after closure, could be promising, as it was found altered after predator-exposure in another *Daphnia* species^40^. Additionally, during initial test recordings with *A. vesiculosa* and random prey from an outdoor pond in the Botanic Garden Freiburg, we filmed a very swift and successful active escape of a copepod out of a triggered trap (Movie S15). The animal’s reaction time was 5 ms. Within a time span of 16 ms it swam out of the closing trap (total closure duration: 57 ms). Estimating a total trap length of ~3 mm (which is at the lower end of the typical length range^3^) and assuming that the animal travels half the length gives a flight response velocity of 0.09375 m/s. This velocity is still comparatively slow to the velocities copepods can reportedly achieve^41^. Because there are no *A. vesiculosa* plants cultivated in the pond where the copepod originated from, its rapid escape cannot be counted as an induced defence, as described before. Apparently, some (groups of) animals are intrinsically capable of escaping the fast snap-traps of the waterwheel plant, although, admittedly, the trap filmed was not very fast compared to others. However, elucidating the neuro-mechanical basis for instantaneous and rapid escapes also constitutes a promising aspect for future studies.

## Materials and Methods

### Plant cultivation, prey animal selection and culture

The plants used for the experiments were healthy tropical ecotypes of *Aldrovanda vesiculosa* and originally collected from Girraween Lagoon, approximately 30 km southwest of Darwin, NT, Australia^11,42^. They were initially provided by Dr. Lubomír Adamec from the living collection of the Institute of Botany of the Czech Academy of Sciences at Třeboň. The plants were grown in aquaria in the tropical greenhouse of the Freiburg Botanic Garden (University of Freiburg, Germany) at ~24 °C, water pH 6.5–7, and under artificial light according to their requirements^43^.

*Daphnia longicephala* (Fig. 1d) were reared in 1 L Weck beakers (J. Weck GmbH und Co. KG, Wehr-Öflingen, Germany) at the Department of Animal Ecology, Evolution and Biodiversity of the University of Bochum, Germany. The animals were cultured in the department for several generations under laboratory conditions (16:8-hour day-night cycle, 20 °C +/− 1 °C). The glass beakers contained charcoal filtered tap water. In the culture and the experiments, animals were fed *ad libitum* with the green algae *Acutodesmus obliquus*. Culture beakers and beakers used in the experiments were cleaned every other day and water was exchanged monthly. All specimens of *D. longicephala* were genetic clones resulting from parthenogenetic reproduction, with the initial clone originating from Lara Pond in Australia^44,45^. Over the course of the experiments, two water-filled 50 ml Falcon tubes with *D. longicephala* were sent daily to the Plant Biomechanics Group (University of Freiburg, Germany). At the Plant Biomechanics Group, daphniids were transferred into larger aquaria filled with tap water and fed with *Acutodesmus*. Prey were between three and six days old at the time of the individual experiments.

### Recordings of prey capture attempts (PCAs)

Prior to the actual recordings of the prey capture attempts (PCAs), the waterwheel plants in the tropical greenhouse of the Freiburg Botanic Garden were transferred into plastic petri dishes filled with tap water (~24 °C) and transferred into the Plant Biomechanics Group microscopy lab. Each seventh trap leaf whorl, as counted beginning from the apical bud, was cut off under water to ensure similar trap ages. Single traps were then carefully cut off under water with micro-scissors without triggering closure. For each experimental attempt, a single, open trap was transferred under water into a polystyrene cuvette (Carl Roth GmbH + Co. KG, Karlsruhe, Germany). The petiolus was grasped with tweezers and embedded in a small piece of modelling clay, which then was carefully pressed against the cuvette wall for fixation. Plant and animal specimens were then transferred into a temperature constant chamber (24 °C) in the Biology II/III building (University of Freiburg, Germany).

For the actual analyses, prey animals were taken from the stock and transferred into the cuvettes by using a plastic pipette. The volume of water and prey inside the cuvettes was kept at 3 ml to facilitate the determination of prey density. PCAs were recorded with 1,000 fps by using a Motion Pro Y4 or a Motion Pro NX4 high-speed camera (Integrated Design Tool (IDT) Inc., Tallahassee, USA) mounted on horizontally aligned stereomicroscopes, either a Wild M420 (Wild Macroscope, Wild Heerbrugg, Switzerland) or an Olympus SZX7 (Olympus Optical Co., Japan). Two cold light sources (Constellation 120 high-performance LEDs, IDT Inc., Tallahassee, USA) were positioned with flexible tripods (Magic Arm Manfrotto 244RC, Manfrotto, Cassola, Italy). The Motion Studio software (v.2.10.05, IDT Inc. Tallahassee, USA) was used for data acquisition. Only PCA recordings, where the prey stayed in the focal plane, were stored and further analysed. PCAs were grouped according to their outcome, i.e. successful capture of prey or unsuccessful trap closure (prey could escape).

### Evaluation of recorded prey capture attempts

From each recorded PCA, the image sequence was further analysed with the software Fiji/ImageJ^46^. An area around the trap margins, with a distance corresponding to the body length of the respective prey animal (without second antennae and tail spine), was defined as the danger zone (DZ). The time-points of prey entering the DZ and the inside of the trap (trap interior, TI) were determined, as well as the respective staying times in these zones. In addition, the frequency, timings, and types of animal movements were investigated: second antennae downstroke (backward directed synchronous movement of the second antennae), second antennae return stroke (forward directed synchronous movement of the second antennae), and asynchronous movement of the second antennae deployed by the animal for changing the swimming direction. In addition, the orientation of the prey in relation to the trap midrib during triggering was also examined, as well as the movement type and body part responsible for triggering trap closure. Additionally, the trap closure duration for each *A. vesiculosa* trap was measured until an opening angle of approx. 0–5° between the trap lobes was achieved and the prey was trapped (see ref. ^11^ for a detailed definition of trap closure stages).

For quantifying prey movements, the contours of the animals during the recorded PCAs were retraced manually at the first frame of the respective recording where the prey animal was visible and then in every 50th frame. The coordinates of the geometric centres of gravity and their individual distances were determined. Then, for each recording the frame was selected in which the prey animal was deepest in the trap (closest to the midrib), which often corresponds to the event of trap triggering. The corresponding coordinate of the geometric centre of gravity was defined as target point (y = 0), whereas the coordinate for the geometric centre of gravity determined for the first frame was defined as starting point (y = 1).

All following calculations were performed with LibreOffice Calc (v.5.1.6.2, LibreOffice contributors). First, the total distances from the defined starting points to the defined target points and the remaining distances of the individual geometric centres of gravity to the target point were calculated, and the results converted into fractions of the total distance (0 ≤ y ≤ 1). If y = 1, then the prey is furthest away from the trap at this time. In the cases in which the prey swam out of the trap and away from it, this distance was also calculated proportionally to the total distance. The relative distance to the trap could be indicated by the data obtained in this way. The data were finally plotted against time. Furthermore, the capture rates of PCAs were calculated, as well as the trap trigger durations (t_trigger_), trap closure durations (t_closure_), and staying durations of prey inside the danger zone (t_DZ_) and inside the respective traps (t_TI_). The triggering time t_trigger_ describes the time span beginning from the introduction of the prey into the respective cuvette until the triggering of the trap took place.

The software GNU R v.1.0.143^47^ was used for statistics. The Shapiro-Wilk test was performed to test results for normal distribution. Different prey dependent variables (prey density, prey movement, prey age) were tested for correlations with plant response measurements (t_trigger_, PCA successful/unsuccessful) as well as prey age with prey movement. The ‘spearman rho correlation tests’ was performed between ordinal data sets as well as the ‘psych’ R-library to perform biserial tests for correlation tests that included the binary data set ‘PCA successful/unsuccessful’.

## Supplementary information

supplementary information

Supplementary Movie 1

Supplementary Movie 2

Supplementary Movie 3

Supplementary Movie 4

Supplementary Movie 5

Supplementary Movie 6

Supplementary Movie 7

Supplementary Movie 8

Supplementary Movie 9

Supplementary Movie 10

Supplementary Movie 11

Supplementary Movie 12

Supplementary Movie 13

Supplementary Movie 14

Supplementary Movie 15

## Data Availability

All data needed to evaluate the conclusions in the paper are present in the paper and/or the electronic supplementary material. Additional data related to this paper may be requested from the authors.
