# Supplementary material for: Prey capture analyses in the carnivorous aquatic waterwheel plant (Aldrovanda vesiculosa L., Droseraceae)
Source: Sci Rep. 2019 Dec 9;9:18590. doi: 10.1038/s41598-019-54857-w (PMC6901478; doi:10.1038/s41598-019-54857-w)
Supplement: Supplementary file 1 — supplementary information [file 41598_2019_54857_MOESM1_ESM.pdf]

SUPPLEMENTARY INFORMATION FOR

Prey capture analyses in the carnivorous aquatic waterwheel plant (*Aldrovanda vesiculosa* L., Droseraceae)

Simon Poppinga[1,2,*], Jassir Smaij[1], Anna Sofia Westermeier[1,3], Sebastian Kruppert[4], Martin Horstmann[4], Ralph Tollrian[4], Thomas Speck[1,2,3,5]

[1]*Plant Biomechanics Group, Botanic Garden, University of Freiburg, Freiburg im Breisgau, Germany.*

[2]*Freiburg Materials Research Center (FMF), University of Freiburg, Freiburg im Breisgau, Germany.*

[3]*Freiburg Center for Interactive Materials and Bioinspired Technologies (FIT), University of Freiburg, Freiburg im Breisgau, Germany.*

[4]*Department of Animal Ecology, Evolution and Biodiversity, Ruhr-University Bochum, Bochum, Germany.*

[5]*Cluster of Excellence livMatS @ FIT Freiburg Center for Interactive Materials and Bioinspired Technologies, University of Freiburg, Freiburg im Breisgau, Germany*

**Corresponding author. Email: simon.poppinga@biologie.uni-freiburg.de*

Legends for Supplementary Movies

Movie S1: Successful prey capture event (PCA) 01. Recording speed: 1,000 fps, playback rate: 20 fps.

Movie S2: Successful prey capture event (PCA) 02. Recording speed: 1,000 fps, playback rate: 20 fps.

Movie S3: Successful prey capture event (PCA) 03. Recording speed: 1,000 fps, playback rate: 20 fps.

Movie S4: Successful prey capture event (PCA) 04. Recording speed: 1,000 fps, playback rate: 20 fps.

Movie S5: Successful prey capture event (PCA) 05. Recording speed: 1,000 fps, playback rate: 20 fps.

Movie S6: Successful prey capture event (PCA) 06. Recording speed: 1,000 fps, playback rate: 20 fps.

Movie S7: Successful prey capture event (PCA) 07. Recording speed: 1,000 fps, playback rate: 20 fps.

Movie S8: Successful prey capture event (PCA) 08. Recording speed: 1,000 fps, playback rate: 20 fps.

Movie S9: Successful prey capture event (PCA) 09. Recording speed: 1,000 fps, playback rate: 20 fps.

Movie S10: Successful prey capture event (PCA) 10. Recording speed: 1,000 fps, playback rate: 20 fps.

Movie S11: Unsuccessful prey capture event (PCA) 11. Recording speed: 1,000 fps, playback rate: 20 fps.

Movie S12: Unsuccessful prey capture event (PCA) 12. Recording speed: 1,000 fps, playback rate: 20 fps.

Movie S13: Unsuccessful prey capture event (PCA) 13. Recording speed: 1,000 fps, playback rate: 20 fps.

Movie S14: Unsuccessful prey capture event (PCA) 14. Recording speed: 1,000 fps, playback rate: 20 fps.

Movie S15: Escape of a copepod out of the triggered *Aldrovanda* trap. Recording speed: 1,000 fps, playback rate: 20 fps.
